# Genomic epidemiology of multidrug-resistant *Klebsiella pneumoniae* reveals lineage-specific resistance patterns with limited virulence determinants

**DOI:** 10.3934/microbiol.2026027

**Published:** 2026-09-04

**Authors:** Aiman Waheed, Sumera Afzal Khan, Taj Ali Khan, Sajjad Ahmad, Aakash Ahmad Khattak, Taane G. Clark, Nigel G. Ternan, Fahad Ahmed, Ibrahim M. Banat, Zahoor Khan

**Affiliations:** 1 Center of Biotechnology and Microbiology, University of Peshawar, Peshawar, Pakistan; 2 Institute of Pathology and Diagnostic Medicine, IPDM, Khyber Medical University, Peshawar, Pakistan; 3 Department of Infection Biology, Faculty of Infectious and Tropical Diseases, London School of Hygiene and Tropical Medicine, London, UK; 4 Faculty of Epidemiology and Population Health, London School of Hygiene and Tropical Medicine, London, UK; 5 Nutrition Innovation Centre for Food and Health (NICHE), School of Biomedical Sciences, Ulster University, Coleraine, N. Ireland, UK; 6 Pharmaceutical Sciences Research Group, School of Biomedical Sciences, Ulster University, Coleraine, N. Ireland, UK

**Keywords:** *Klebsiella pneumoniae*, genomic epidemiology, antimicrobial resistance, whole-genome sequencing, multidrug resistant

## Abstract

The emergence of multidrug-resistant (MDR) *Klebsiella pneumoniae* undermines treatment efficacy, while limited genomic epidemiological data constrain understanding of local transmission dynamics. We therefore aimed to investigate the genomic epidemiology and the antimicrobial resistance landscape of *K. pneumoniae* in tertiary-care settings in Peshawar, Pakistan. A total of 497 clinical samples (urine, blood, sputum, pus, wound swabs, sterile body fluids) were collected from 01 August 2024 to 31 July 2025. *K. pneumoniae* was identified using the API 20E kit, followed by antimicrobial susceptibility testing (AST), with colistin and tigecycline susceptibility determined by broth microdilution. Whole-genome sequencing (WGS) was carried out on 43 MDR and XDR isolates to resolve clonal diversity, resistome composition, virulence-associated loci, and phylogenetic relationships. Among 497 samples, 152 *K. pneumoniae* isolates were identified, exhibiting widespread resistance to β-lactams, cephalosporins, fluoroquinolones, aminoglycosides, and carbapenems, while colistin and tigecycline retained substantial *in vitro* activity. Overall, 37% and 24% of isolates were classified as MDR and XDR, respectively. Forty-three MDR/XDR isolates were subjected to WGS, of which 34 high-quality *K. pneumoniae* genomes passed quality control and species verification for downstream analysis. Genomic analysis identified 18 sequence types, with ST147 (7/34, 20.6%), ST37 (4/34, 11.8%), ST2629 (3/34, 8.8%), ST39 (3/34, 8.8%), and ST870 (2/34, 5.9%) representing the predominant lineages. The dominant high-risk clones, particularly ST147, ST11, ST231, and ST42-3LV, harbored multiple antimicrobial resistance determinants, including *bla*CTX-M-15 (20/34) or *bla*CTX-M-14 (2/34), *bla*KPC-2 (2/34), *blaOXA*-48 (1/34), *blaOXA*-181 (1/34), *bla*NDM-5 (1/34), aminoglycoside-modifying enzymes, fluoroquinolone resistance determinants, porin disruptions (ompK35/ompK36; 9/34), and colistin resistance–associated mutations in *pmrB* in 4 isolates. ST147 was the predominant lineage and was consistently associated with the KL64 capsule type and O1/O2-associated O-loci. Virulence profiling demonstrated a predominantly low-virulence population, with 19/34 (55.9%) isolates carrying yersiniabactin as the only detected virulence determinant, whereas aerobactin, colibactin, and *rmpA/rmpA2* were absent from all genomes. Collectively, these findings demonstrate that MDR *K. pneumoniae* circulating in Peshawar are dominated by internationally disseminated high-risk clones characterized by extensive antimicrobial resistance but limited virulence potential, underscoring the importance of genomic surveillance to strengthen infection prevention and antimicrobial stewardship.

## 1. Introduction

*Klebsiella pneumoniae* has emerged as one of the most clinically important Gram-negative pathogens worldwide because of its remarkable capacity to acquire antimicrobial resistance (AMR) and cause a broad spectrum of healthcare- and community-associated infections, including urinary tract infections (UTIs), pneumonia, bloodstream infections, and soft tissue infections, particularly among critically ill and immunocompromised patients [1]. The rapid emergence and global dissemination of multidrug-resistant (MDR) and carbapenem-resistant *K. pneumoniae* have led the World Health Organization (WHO) to classify this pathogen among the highest-priority bacterial threats requiring urgent surveillance and therapeutic intervention [2]. The growing public health importance of *K. pneumoniae* is driven by the convergence of AMR and virulence. Traditionally, *K. pneumoniae* has been categorized into two major pathotypes: The classical *K. pneumoniae* (cKp), predominantly associated with nosocomial infections and extensive AMR, while the hypervirulent *K. pneumoniae* (hvKp), historically linked to community-acquired invasive infections in healthy individuals [2],[3]. The cKp strains commonly harbor extended-spectrum β-lactamases (ESBLs) and carbapenemases, while hvKp strains are characterized by enhanced virulence determinants, including siderophore systems and hypermucoviscosity regulators, which facilitate rapid dissemination and metastatic infections [4]. Moreover, convergence of carbapenem resistance and hypervirulence has resulted in the emergence of carbapenem-resistant hypervirulent *K. pneumoniae* (CR-hvKp), representing a major global public health concern [5],[6].

The prevalence of high-risk *K. pneumoniae* lineages is strongly influenced by antimicrobial resistance mechanisms and genomic determinants associated with persistence and transmission. Surface polysaccharides, including capsular polysaccharides (K-locus) and lipopolysaccharide O-antigens (O-locus), contribute to immune evasion and host adaptation, while specific capsular types such as KL64 have been closely associated with globally disseminated carbapenem-resistant clones [7]–[9]. In parallel, whole-genome sequencing (WGS) has transformed molecular epidemiology by enabling high-resolution characterization of sequence types (STs), resistance genes, virulence loci, mobile genetic elements, and phylogenetic relationships that cannot be resolved using conventional phenotypic or targeted molecular approaches [10],[11]. Carbapenem resistance in *K. pneumoniae* is primarily mediated by the acquisition of carbapenemase genes such as *bla*KPC, *bla*NDM, and *blaOXA*-48 like variants, often in combination with porin loss and other chromosomal mutations that further reduce antibiotic susceptibility [12],[13]. The international dissemination of these determinants is largely driven by successful high-risk clones and horizontal plasmid transfer [14].

The burden of MDR and extensively drug-resistant (XDR) *K. pneumoniae* is particularly severe in low- and middle-income countries such as Pakistan, where inappropriate antimicrobial use, inadequate infection prevention practices, and limited genomic surveillance have accelerated the emergence and spread of resistant lineages [15]. Although *K. pneumoniae* is a major cause of healthcare-associated infections in Pakistan, with mortality approaching 40–70% in severe infections [16], current surveillance has relied predominantly on phenotypic AST or targeted PCR-based detection of selected resistance genes [17],[18]. Consequently, the genomic diversity, population structure, high-risk international clones, virulence repertoire, and evolutionary relationships of circulating *K. pneumoniae* remain poorly characterized, particularly in northwestern Pakistan. Furthermore, it remains unclear whether the expansion of MDR/XDR *K. pneumoniae* in this region is driven primarily by dissemination of established international high-risk clones, local clonal expansion, acquisition of mobile resistance determinants, or convergence of resistance with hypervirulence [17]. Addressing these knowledge gaps is essential for strengthening genomic surveillance, guiding infection prevention strategies, and informing antimicrobial stewardship programs.

We hypothesized that MDR and XDR *K. pneumoniae* circulating in tertiary-care hospitals in Peshawar are dominated by internationally disseminated high-risk lineages carrying extensive drug resistance determinants but a comparatively limited virulence repertoire. This hypothesis was rigorously tested through integrated phenotypic and genomic analyses and was strongly supported by our findings. WGS, coupled with epidemiological and AST data, confirmed the predominance of internationally disseminated high-risk clones characterized by an extensive resistome and a relatively restricted virulome. These findings provide robust genomic epidemiological evidence to support the implementation of genomic surveillance, infection prevention and control measures, and antimicrobial stewardship programs for the containment of high-risk *K. pneumoniae* lineages in Pakistan.

## 2. Materials and methods

### 2.1. Study design, ethical approval, and sample collection

This cross-sectional study was conducted between 01 August 2024 and 31 July 2025 using clinical specimens collected from patients attending three major tertiary care hospitals in Peshawar, Pakistan: Khyber Teaching Hospital (KTH), Hayatabad Medical Complex (HMC), and Lady Reading Hospital (LRH). The three participating hospitals were selected because they serve as the principal public tertiary referral centers for Peshawar and receive patients from multiple surrounding districts, thereby providing broad regional representation. Ethical approval was obtained from the Research Ethics Board, University of Peshawar (Approval No. REB-10; approved on 12 July 2024). Written informed consent was obtained from all participants or their legal guardians before specimen collection, in accordance with institutional and international ethical guidelines.

A total of 497 non-duplicate samples (urine, blood, sputum, pus, wound swabs, and sterile body fluids) were collected from patients with clinical features suggestive of Gram-negative bacterial infection. To minimize confounding by prior antimicrobial exposure, patients who had received systemic antibiotic therapy within 72 h before specimen collection were excluded. Demographic and clinical metadata, including age, sex, hospital, district of residence, specimen type, and clinical diagnosis or site of infection, were retrieved from standardized laboratory request forms and the hospital laboratory information system at the time of specimen collection. These variables were subsequently integrated with microbiological and genomic data for epidemiological analyses.

### 2.2. Isolation and API 20E based confirmation of *K. pneumoniae*

Clinical specimens were processed using standard microbiological techniques. Samples were inoculated onto blood agar, CLED, and MacConkey agar, and incubated aerobically at 37 °C for 18–24 h. The *K. pneumoniae* isolates were identified based on their characteristic large, mucoid, lactose-fermenting pink colonies on MacConkey agar, and Gram-negative rod-shaped appearance on Gram staining. Isolates were characterized using conventional biochemical tests (citrate utilization, urease, indole, methyl red, Voges–Proskauer, oxidase, catalase, and motility), followed by confirmation using the API 20E identification system (bioMérieux, France) with biochemical profiles concordant with the *K. pneumoniae* reference identification code 5214573. Because biochemical identification systems may occasionally misclassify members of the *K. pneumoniae* species complex, species identity of all sequenced isolates was independently verified by whole-genome sequencing using Kraken2 taxonomic classification before downstream genomic analyses.

### 2.3. Antimicrobial susceptibility profiling and resistance classification

AST was performed on all confirmed 152 *K. pneumoniae* isolates using the standard Kirby-Bauer disk diffusion method on Mueller–Hinton agar (MHA) using CLSI M100 criteria (34th Edition) [19],[20]. The antibiotic panel included AMC, TZP, CZ, FOX, CAZ, CTX, FEP, ETP, MEM, AK, CN, TOB, CIP, LEV, TET, SXT, and TGC. Nitrofurantoin testing was performed only for urinary isolates [21]. Colistin and tigecycline MICs were determined by broth microdilution using a commercial ComASP™ Colistin assay (0.25–16 µg/mL). Based on resistance profiles, MDR and XDR phenotypes were classified in accordance with the standardized interim definitions jointly proposed by the European Centre for Disease Prevention and Control (ECDC) and the U.S. Centers for Disease Control and Prevention (CDC), whereby MDR denotes resistance to at least one agent in three or more antimicrobial classes, and XDR denotes resistance to at least one agent in all but two or fewer antimicrobial classes [22].

### 2.4. Phenotypic assessment of hypermucoviscosity and hemolytic activity

Hypermucoviscosity was assessed using the string test. A viscous string ≥5 mm formed by stretching colonies from blood agar plates was considered positive [23]. Hemolytic activity was evaluated on 5% sheep blood agar after incubation at 37 °C for 24–48 h [24].

### 2.5. Whole-genome sequencing and bioinformatics analysis

A purposive subset of 43 *K. pneumoniae* isolates was selected for WGS to maximize representation of clinically important antimicrobial resistance phenotypes while maintaining analytical feasibility. Isolates were selected based on MDR and XDR phenotypes, hypermucoviscosity phenotype, specimen source, hospital of origin, and genomic DNA quality to capture the phenotypic and epidemiological diversity of the isolate collection. Genomic DNA was extracted using the GeneJET Genomic DNA Purification Kit and sequenced on the Illumina MiSeq platform (paired-end 150 bp, ~50× target coverage) [25]. The quality of raw reads was assessed using FastQC (v0.11.9), followed by trimming of poor-quality reads via Trimmomatic (v0.36) [26]. Species confirmation and contamination screening were performed using Kraken2 software with visualization in Krona [27]. High-quality reads were assembled de novo using SPAdes, and assembly quality was evaluated using QUAST and CheckM2, including assessment of assembly continuity, genome completeness, and contamination estimates. Only assemblies meeting acceptable quality metrics were retained for downstream analyses [28]. Genome annotation was carried out using Prokka and BV-BRC pipelines. MLST, capsular (K-locus) and O-antigen typing, and virulence and resistance scoring were performed using Kleborate (v2.3.2) [29].

### 2.6. Phylogenomic analysis to check diversity

Phylogenetic relationships were inferred using the BV-BRC Codon Tree pipeline, based on 100 conserved single-copy genes. Maximum-likelihood trees were generated using RAxML with 100 bootstrap replicates. Trees were visualized using iTOL v7 [30].

## 3. Results

### 3.1. Epidemiological and clinical profile of *K. pneumoniae* isolates in tertiary-care hospitals

Between August 2024 and July 2025, a total of 497 clinical specimens were analyzed, of which 152 (30.6%) were confirmed *K. pneumoniae* via Gram stain and API (20E). Among the 152 non-duplicate isolates, KTH and LRH each contributed 52 isolates (34.2%), whereas Hayatabad Medical Complex contributed 48 isolates (31.6%). Most isolates originated from Mardan (34.2%), followed by Peshawar (28.3%), Charsadda (23.0%), Swabi (9.9%), and other surrounding districts (4.6%). The predominance of isolates from densely populated districts may reflect higher patient referral rates to tertiary-care hospitals. Urine was the most common specimen source (n = 52, 34.2%), followed by pus (n = 33, 21.7%), blood (n = 26, 17.1%), and sputum (n = 20, 13.2%), underscoring the involvement of *K. pneumoniae* in UTI, soft tissue, and bloodstream infections. A slight male predominance was observed, 81 (53.3%), and infections were most frequent among adults aged 41–60 years, 49 (32.2%), followed by those older than 60 years, 34 (22.4%), and 21–40 years, 33 (21.7%). Pediatric cases accounted for a relatively smaller proportion of 13 (8.6%), indicating a comparatively lower disease burden in this age group (Figure 1).

**Figure 1.**
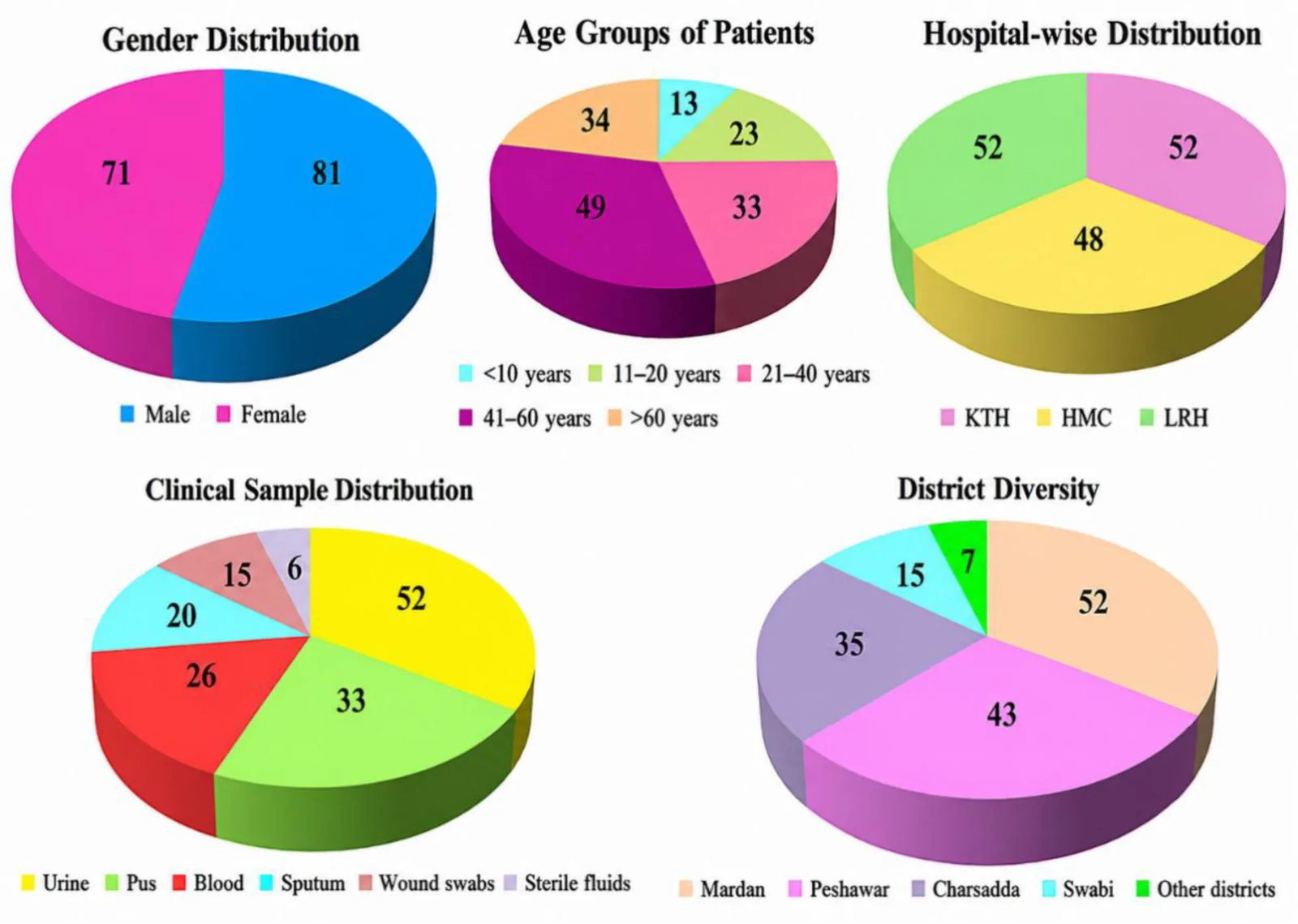
Demographic and clinical features of *K. pneumoniae* isolates from urine, blood, sputum, pus, wound swabs, and sterile body fluids.

### 3.2. Antimicrobial susceptibility and resistance trends in *K. pneumoniae*

The AST results demonstrated high resistance rates against β-lactam antibiotics. Resistance to amoxicillin–clavulanic acid was detected in 120 (78.9%), while piperacillin–tazobactam resistance was observed in 58 (38.2%). Among cephalosporins, resistance was highest to cefazolin 127 (83.6%), followed by ceftriaxone 104 (68.4%), ceftazidime 100 (65.8%), cefepime 91(59.9%), and cefoxitin 112 (73.7%), indicating widespread resistance to extended-spectrum cephalosporins. Carbapenems showed comparatively better activity; ertapenem resistance was detected in 60(39.5%), while meropenem resistance was observed in 58 (38.2%) isolates. Moderate susceptibility was observed among aminoglycosides. Resistance rates were 63 (41.4%) for amikacin, 67 (44.1%) for gentamicin, and 74 (48.7%) for tobramycin. Fluoroquinolone activity was limited, with resistance to ciprofloxacin observed in 82 (53.9%) isolates and resistance to levofloxacin in 70 (46.1%) isolates. Tetracycline resistance was detected in 62 (40.8%) isolates, while trimethoprim–sulfamethoxazole showed poor activity, with resistance observed in 87 (57.2%) isolates. Tigecycline exhibited excellent activity, with 139 (91.4%) isolates remaining susceptible.

Colistin demonstrated the highest *in vitro* activity, with 121 isolates (79.6%) classified as susceptible. Colistin susceptibility was determined using MIC testing, and all resistant isolates exhibited MIC values ≥4 µg/mL. Nitrofurantoin and fosfomycin susceptibility tests were performed exclusively on urinary isolates (n = 52). Among these, resistance to nitrofurantoin was observed in 18 (34.6%) isolates, while fosfomycin resistance was detected in 11 (21.2%) isolates (Figure 2). Based on ECDC/CDC definitions, 27 (17.8%) isolates were fully susceptible to all tested antimicrobial classes; 33 (21.7%) isolates exhibited resistance to one or two classes; 56 (36.8%) isolates were classified as MDR, and 36 (23.7%) isolates as XDR.

**Figure 2.**
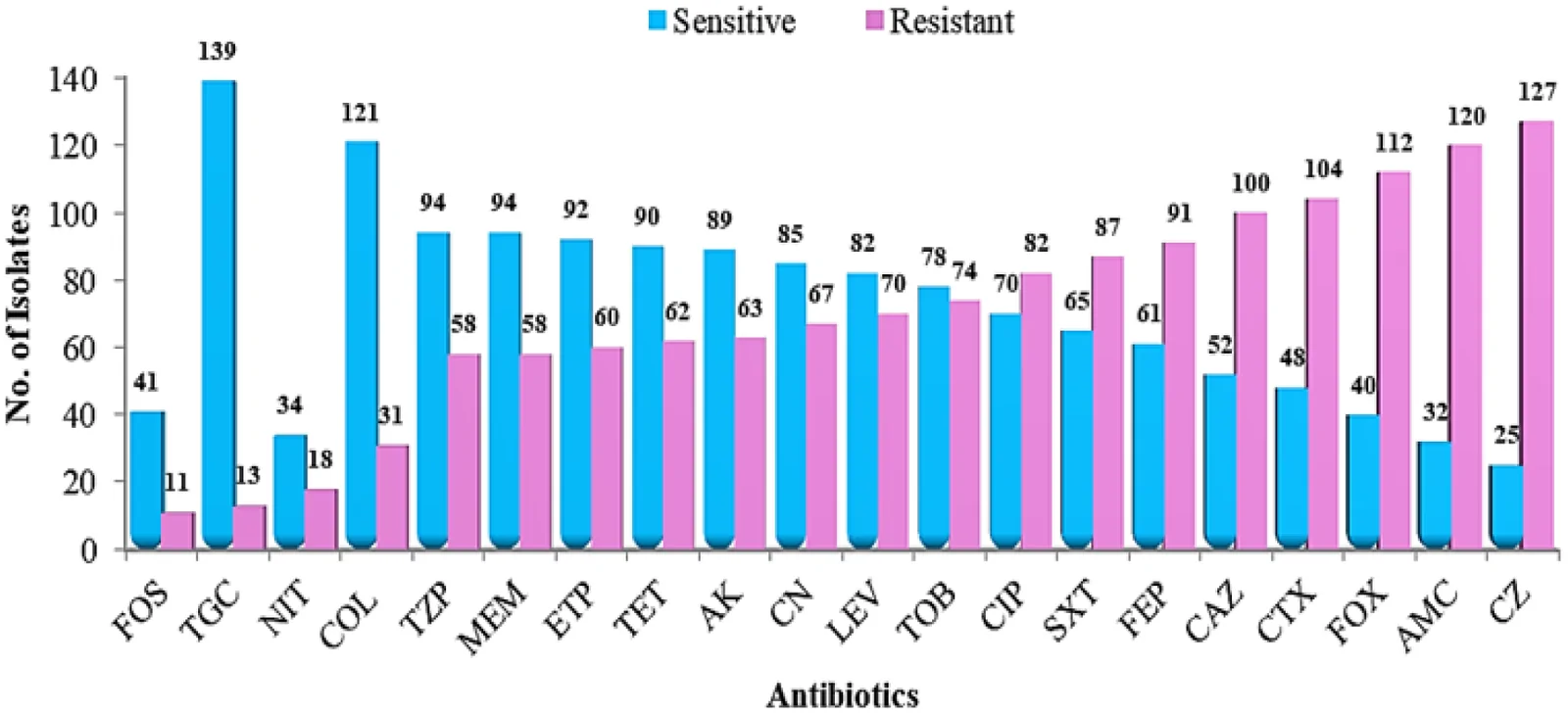
AST and resistance patterns of *K. pneumoniae* isolates (n = 152). AST was performed using the Kirby–Bauer disk diffusion method on MHA according to the CLSI M100, 34th Edition guidelines. Colistin (COL) and tigecycline (TGC) susceptibility were determined by broth microdilution. Nitrofurantoin (NIT) and fosfomycin (FOS) susceptibilities were performed exclusively for urinary isolates (n = 52) in accordance with CLSI/EUCAST recommendations. Antibiotic abbreviations: AMC, amoxicillin–clavulanic acid; TZP, piperacillin–tazobactam; CZ, cefazolin; FOX, cefoxitin; CAZ, ceftazidime; CTX, ceftriaxone; FEP, cefepime; ETP, ertapenem; MEM, meropenem; AK, amikacin; CN, gentamicin; TOB, tobramycin; LEV, levofloxacin; CIP, ciprofloxacin; TET, tetracycline; SXT, trimethoprim–sulfamethoxazole; TGC, tigecycline; COL, colistin; NIT, nitrofurantoin; and FOS, fosfomycin.

### 3.3. Overview of WGS of *K. pneumoniae* and its population structure

WGS was performed on 43 selected MDR and XDR isolates. Species verification using Kraken2 confirmed that 34 isolates (79.1%) were *K. pneumoniae*. The remaining genomes were identified as *Klebsiella quasipneumoniae* subsp. *quasipneumoniae* (n = 1) or showed evidence of contamination with *Escherichia coli* (n = 4), *Pseudomonas aeruginosa* (n = 3), and *Enterobacter cloacae* (n = 1).

### 3.4. Phylogenomic distribution and clinical epidemiological characteristics of *K. pneumoniae* genomes

The phylogenetic relationship of 34 *K. pneumoniae* genomes, incorporated with demographic, clinical, geographic, and hypermucoviscsity attributes, is represented in Figure 3. The maximum-likelihood phylogeny demonstrates clear lineage structuring, with multiple genetically related clusters. Hypermucoid and non-hypermucoid phenotypes were dispersed across phylogeny, indicating that hypermucoviscosity is not lineage-restricted but arises across genetic backgrounds. Hypermucoid isolates constituted a minority and were interspersed among predominantly non-hypermucoid strains. Clinically, most isolates were recovered from pus samples and were associated with soft-tissue infections, including abscesses, wounds, and cellulitis, whereas a smaller number were recovered from urine or blood and were linked to UTIs or septicemia. The isolates were obtained from patients of both sexes across a broad age range and originated from multiple districts, including Mardan, Peshawar, Swabi, Charsadda, and Takht Bhai, with no strict geographic clustering evident within the phylogeny (Figure 3).

**Figure 3.**
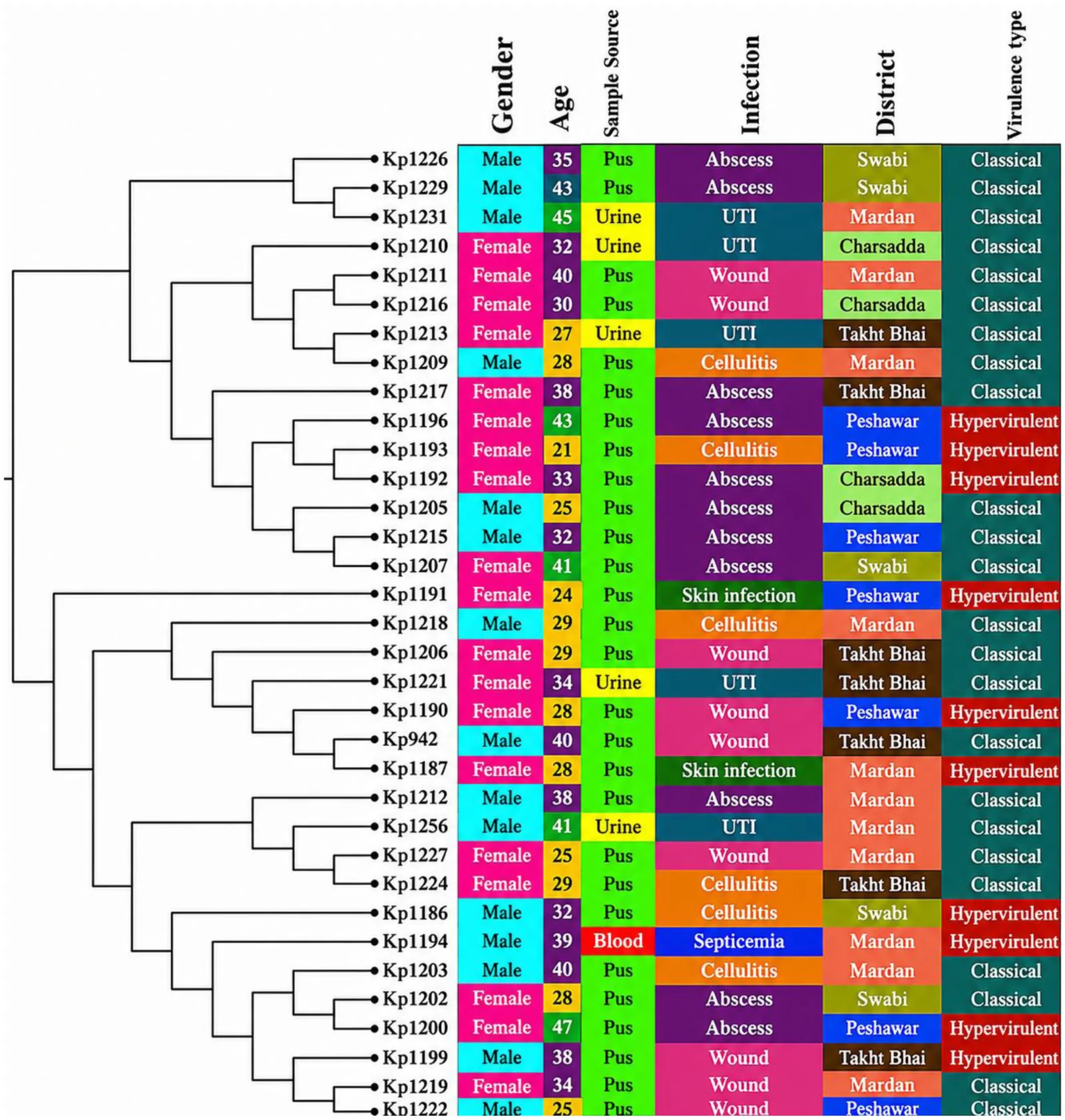
Genome-wide phylogenetic tree for the 34 *Klebsiella pneumoniae* isolates. The tree was annotated using the Interactive online tool ‘Tree of Life’ (iTOL) with gender, age, sample origin, infection, geographic location, and virulence type.

### 3.5. Genome assembly quality and structural diversity of sequenced *K. pneumoniae* isolates

Genome assembly metrics for the 34 WGS *K. pneumoniae* isolates demonstrated substantial variation in assembly quality and structural organization (Table 1). The number of contigs ranged from 7 to 21,518, with most genomes assembled into fewer than 300 contigs, indicating generally good assembly continuity. Two isolates (Kp1216 and Kp1218) exhibited highly contiguous assemblies with only 7 contigs each, whereas a small number of assemblies showed marked fragmentation. Assembly contiguity, as reflected by N50 values, varied widely from 500 bp to 5.33 Mb. Several isolates showed high-quality assemblies with N50 values exceeding 400 kb, suggesting robust genome reconstruction. The size of the largest contig ranged from 38.3 kb to 5.33 Mb, further highlighting differences in assembly completeness across isolates. Total assembled genome sizes ranged from 5.26 Mb to 11.94 Mb. Most genomes clustered between 5.3 and 5.9 Mb, consistent with the expected chromosomal size of *K. pneumoniae* (Table 1).

**Table 1.** Assembly and QC metrics for sequenced *K. pneumoniae* genomes (n = 34).

Strain	Contigs (n)	N50 (bp)	Largest contig (bp)	Total genome size (bp)	GC content (%)
Kp1210	128	287,843	1,370,207	5,717,468	56.9
Kp1215	170	448,857	1,430,174	5,553,382	57.2
Kp1221	143	197,431	604,251	5,498,315	57.1
Kp1203	224	235,629	441,147	5,954,829	56.6
Kp1196	89	373,249	864,557	5,405,795	57.2
Kp1193	99	376,578	840,220	5,423,902	57.2
Kp1192	82	442,842	842,383	5,405,504	57.2
Kp1191	251	238,045	453,936	5,650,939	57.1
Kp1190	158	350,969	760,496	5,369,118	57.4
Kp1211	256	245,379	538,299	6,876,792	57.4
Kp1187	154	350,969	674,180	5,368,084	57.4
Kp1222	233	232,237	492,367	5,953,677	56.7
Kp1224	630	127,939	375,960	6,443,909	58.2
Kp1199	264	183,179	480,908	5,959,172	56.6
Kp1219	236	258,796	786,124	5,443,779	56.8
Kp1186	136	451,750	773,225	5,260,495	57.5
Kp1200	256	214,633	492,459	5,957,613	56.6
Kp1218	148	136,424	366,030	5,990,291	56.8
Kp1216	251	214,633	492,459	5,957,613	57.5
Kp1256	403	146,129	513,349	6,237,651	57.4
Kp1231	240	220,756	419,845	5,626,701	57.1
Kp1229	212	216,442	419,698	5,621,746	57.1
Kp1226	221	275,827	420,007	5,621,275	57.1
Kp1217	745	107,582	300,466	6,298,127	56.7
Kp1213	95	395,886	941,060	5,410,365	57.2
Kp1212	88	352,802	859,020	5,479,812	57.3
Kp1209	97	453,136	941,060	5,411,488	57.3
Kp1207	173	398,981	858,998	5,489,782	57.3
Kp1206	218	501,921	981,321	5,556,869	57.3
Kp1205	218	406,469	1,619,838	5,690,287	57.5
Kp942	193	350,969	1,073,600	5,491,155	57.2
Kp1202	258	214,758	435,873	5,958,915	56.7
Kp1194	103	267,654	606,914	5,700,694	56.9
Kp1227	399	239,143	493,342	5,944,267	57.8

### 3.6. Surface antigen distribution and genetic diversity of *K. pneumoniae* genomes

The 34 genomes comprise 18 distinct sequence types (STs). ST147 was the most dominant lineage 7 (20.6%), followed by ST37: 4 (11.8%), ST2629: 3 (8.8%), and ST39: 3 (8.8%). Several STs, including ST1391-1LV, ST45, and ST870, appeared twice, while the remaining STs (ST11, ST1207, ST1310, ST151, ST231, ST2450-1LV, ST3154, ST42-3LV, ST45-1LV, ST5120, and ST540) appeared once. Capsular (K-locus) typing identified more than 20 distinct KL types, underscoring extensive capsular diversity. KL64 was the most frequent capsular type 7 (20.6%) and was exclusively associated with ST147. Other persistent K-loci included KL24 and KL16, each occurring 3 times (8.8%), while the remaining KL types occurred sporadically across STs. O-antigen analysis demonstrated predominance of O1/O2-associated serogroups, particularly O1/O2v1, which accounted for 21 (61.8%) isolates. O1/O2v2 was detected in 6 (17.6%) isolates, while other less frequent O-loci included O3b 4 (11.8%), O3/O3a 2 (5.9%), and O4 1 (2.9%) (Figure 4).

**Figure 4.**
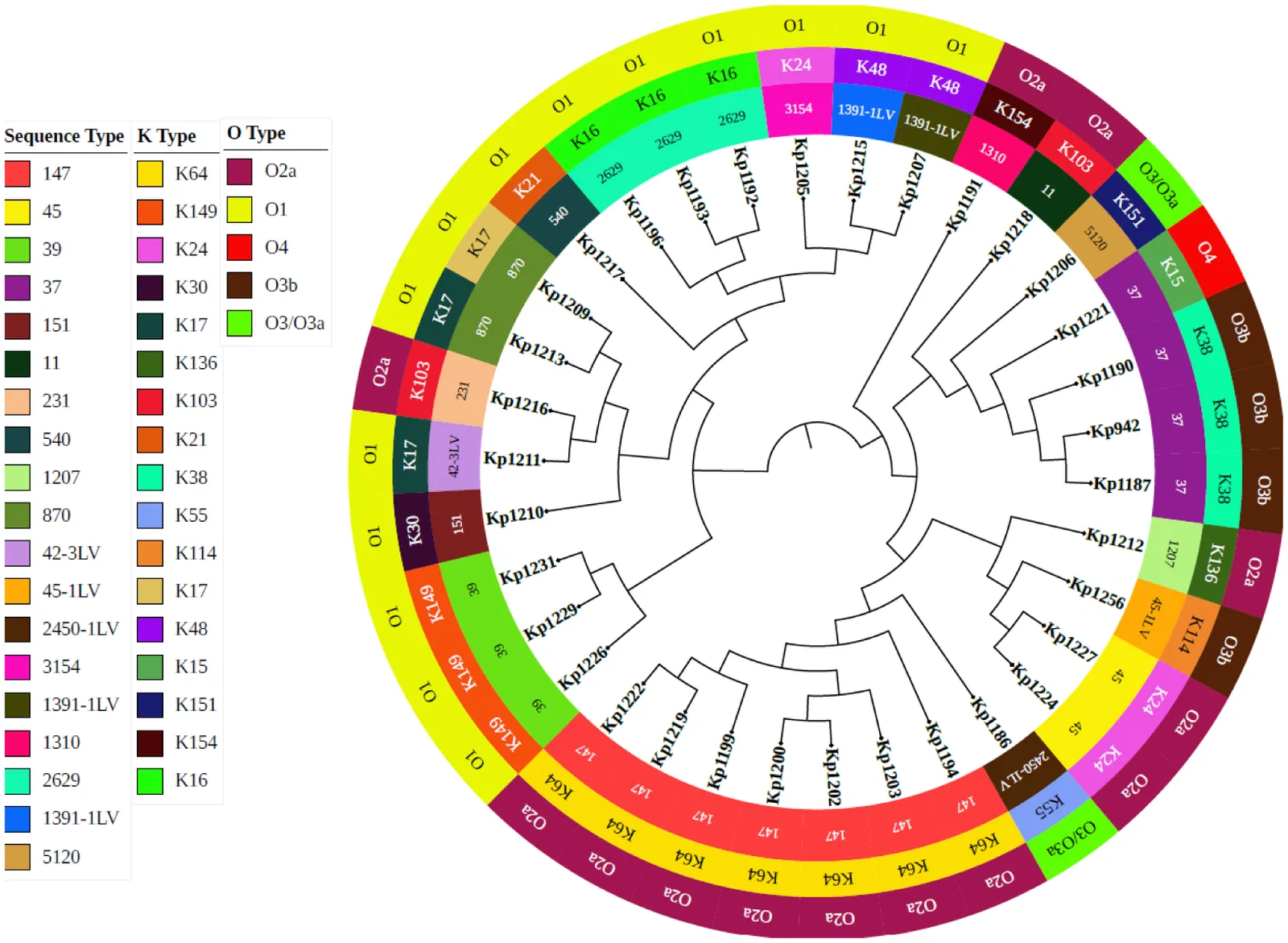
Genome-wide phylogenetic tree for the 34 *Klebsiella pneumoniae* isolates. The tree was annotated using the Interactive Tree of Life online tool (iTOL) with ST, KL, and OL serotypes. The outer layer represents the O types, the second layer shows the K-locus, and the STs are displayed in the innermost layer.

### 3.7. Lineage-specific distribution of yersiniabactin and low virulence profiles in *K. pneumoniae*

Virulence profiling based on Kleborate classification demonstrated a uniformly low virulent background across the *K. pneumoniae* study isolates. Out of 34, 19 (55.9%) isolates carried yersiniabactin (ybt) alone, corresponding to a virulence score 1, while 15 (44.1%) isolates lacked all screened virulence loci and were assigned a virulence score 0. Importantly, no isolate harbored key hypervirulence-associated determinants, including aerobactin (iuc), colibactin (clb), or regulators of hypermucoviscosity (rmpA/rmpA2). Multiple ybt lineages were identified, each associated with distinct integrative conjugative elements (ICEKp). ST147, the most prevalent lineage, consistently carried ybt lineage 16 embedded within ICEKp12, frequently in truncated or incomplete forms, and was associated with YbST variants centered around YbST 280. Other globally relevant lineages also harbored ybt, including ST11 (ybt9; ICEKp3), ST231 (ybt14; ICEKp5), and ST870 (ybt13; ICEKp2), indicating acquisition of siderophore systems across diverse genetic backgrounds. Several STs, including ST37, ST45, ST1207, ST1310, and ST45-1LV, carried ybt10 linked to ICEKp4, whereas ST42-3LV harbored ybt13 associated with an incomplete ICEKp2 element. In contrast, ST39, ST2629, ST2450-1LV, ST540, ST5120, ST3154, and ST1391-1LV consistently lacked ybt, reflecting a complete absence of this virulence determinant within these lineages (Figures 5 and 6).

**Figure 5.**
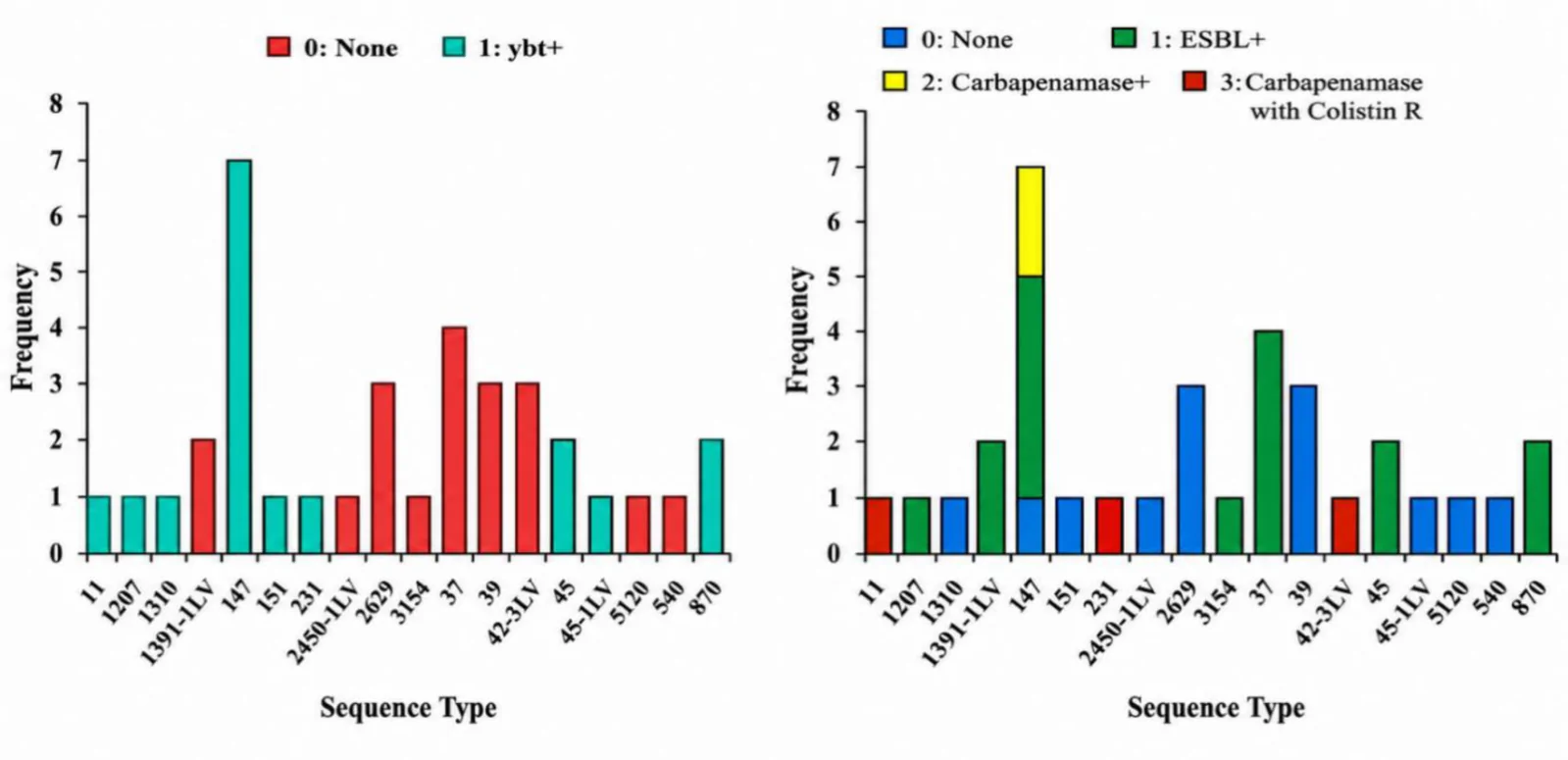
Distribution of Kleborate-defined virulence and antimicrobial resistance scores across *Klebsiella pneumoniae* STs (n = 34). Stacked bar plots show the frequency of resistance scores and virulence scores (left; absence of virulence loci or presence of yersiniabactin alone) and (right; none, ESBL, carbapenemase, carbapenemase with colistin resistance) across STs.

### 3.8. Differential distribution of acquired and chromosomal resistance mechanisms among *K. pneumoniae* STs

The AMR profiles varied markedly across STs. ESBL determinants were detected across STs, indicating widespread dissemination of ESBL-mediated resistance. Carbapenemase genes and colistin resistance, however, were confined to a limited number of STs, reflecting lineage-specific acquisition of high-risk resistance mechanisms. Notably, STs like ST147, ST231, ST42-3LV, and ST11 displayed higher resistance scores and carried multiple acquired genes conferring resistance to β-lactams, aminoglycosides, fluoroquinolones, sulphonamides, tetracyclines, and trimethoprim. These STs frequently harbored ESBL genes (predominantly *bla*CTX-M-15 or *bla*CTX-M-14), often in combination with carbapenemase genes such as *blaOXA*-48, *bla*KPC-2, *blaOXA*-181, or *bla*NDM-5, and mutations in the *pmrB* gene associated with colistin resistance. Several STs such as ST 39, ST2629, ST 24501LV, ST 1310, ST5120, ST540, and ST45-1LV exhibited a resistance score of 0, indicating the absence of acquired ESBL, carbapenemase, and colistin resistance determinants despite their phenotypic antimicrobial resistance profiles (Figures 5 and 6). Aminoglycoside resistance was mediated by genes such as *aac(6′)-Ib*, *rmtB*, and *rmtF*, while fluoroquinolone resistance was associated with plasmid-mediated determinants (*qnrB*, *qnrS*) and chromosomal mutations in *GyrA* (Ser83 substitutions) and *ParC* (Ser80 substitutions). Several carbapenemase-producing isolates also exhibited outer membrane porin disruptions, including partial deletions or truncations of *ompK35* and *ompK36*, further contributing to reduced carbapenem susceptibility.

**Figure 6.**
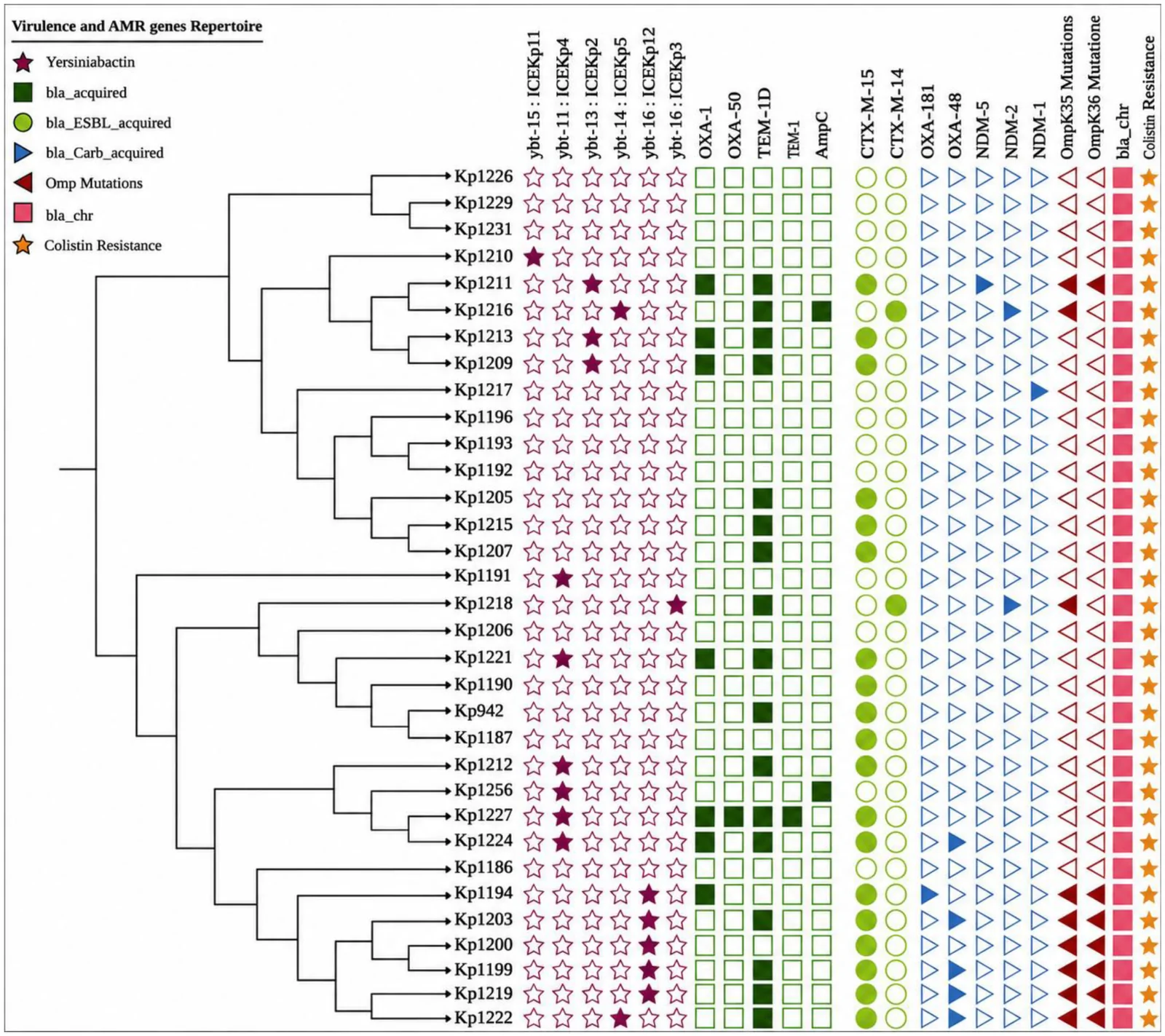
Phylogenomic distribution of virulence and antimicrobial resistance determinants among *Klebsiella pneumoniae* isolates (n = 34). A maximum-likelihood phylogenetic tree is shown, annotated using the Interactive Tree of Life (iTOL). Virulence profiles are defined using Kleborate and include the presence of yersiniabactin (*ybt*) and associated integrative conjugative elements (ICEKp lineages). AMR determinants include acquired and chromosomal β-lactamases (including ESBLs), acquired carbapenemase genes, and markers of colistin resistance indicated by mutations in the *pmrB* gene.

### 3.9. Phylogenomic relationships of our study isolates with national and global isolates

Phylogenomic analysis positioned the *K. pneumoniae* isolates within an evolutionarily variable context, demonstrating that they are closely related to strains from Pakistan and to globally circulating isolates. Notably, several of our isolates grouped tightly with Pakistani reference genomes collected between 2010 and 2020, suggesting long-term establishment and local expansion of these lineages within the country. Furthermore, the distribution of study isolates across branches highlights substantial intra-regional diversity, consistent with repeated introductions and parallel evolution within hospital settings (Figure S1). In the global context, the isolates in the study were interspersed among international strains from Asia, the Middle East, and Europe, rather than forming a geographically restricted cluster. Study isolates showed close phylogenetic proximity to genomes from countries, including China, India, Japan, Turkey, Thailand, Saudi Arabia, and South Korea, reflecting the global dissemination of shared *K. pneumoniae* lineages (Figure S2). This intermingling underscores that the circulating strains in Peshawar are part of globally distributed clonal backgrounds, rather than unique or regionally confined variants.

## 4. Discussion

This study provides an integrated phenotypic and genomic characterization of clinical *K. pneumoniae* isolates circulating in tertiary-care hospitals in Peshawar, Pakistan, and contributes important baseline genomic epidemiological data from a region where WGS based surveillance remains limited. Rather than describing isolated resistance mechanisms, the integration of antimicrobial susceptibility testing with comparative genomics enabled assessment of the population structure, lineage-specific resistance profiles, and phylogenetic relationships of circulating MDR clones. Collectively, these findings strengthen our understanding of the molecular epidemiology of *K. pneumoniae* in Pakistan and provide a framework for future genomic surveillance programs.

The observed prevalence of *K. pneumoniae* among processed clinical specimens is consistent with its recognized role as one of the leading causes of healthcare-associated Gram-negative infections in tertiary-care settings. Differences in prevalence between studies should be interpreted cautiously because they are influenced by hospital type, patient demographics, specimen selection, local antimicrobial prescribing practices, and infection-prevention measures rather than reflecting true geographic variation alone [31],[32]. Environmental factors and healthcare infrastructure have also been proposed as contributors to regional differences in *K. pneumoniae* epidemiology [33].

The predominance of isolates recovered from urine and pus specimens is consistent with the established clinical spectrum of *K. pneumoniae*, particularly its importance in urinary tract and wound infections [31],[34],[35]. However, this specimen distribution should be interpreted in the context of the study design. The isolates originated from routine clinical submissions received at three tertiary-care referral hospitals rather than from a predefined specimen-based sampling framework. Consequently, urinary and wound specimens were proportionally more frequent than bloodstream isolates, reflecting routine hospital case mix rather than differential biological adaptation of specific lineages. Similarly, the observed demographic trends, including the predominance among middle-aged and older adults, are broadly consistent with other reports and likely reflect the higher burden of comorbid conditions, healthcare exposure, and antimicrobial use in these patient populations [35]–[37].

The AST profile observed in this study highlights the continued expansion of resistance among clinically important antimicrobial classes. High resistance to β-lactams and extended-spectrum cephalosporins is consistent with the widespread dissemination of extended-spectrum β-lactamase producing *K. pneumoniae* described across South Asia and other high-burden settings [35],[38]. Continued erosion of cephalosporin activity has important clinical implications because these agents remain widely prescribed for empirical treatment of severe Gram-negative infections [38],[39]. The persistence of elevated resistance across β-lactam subclasses further emphasizes the selective pressure imposed by prolonged antimicrobial exposure within tertiary-care hospitals.

Although aminoglycosides and carbapenems retained comparatively greater *in vitro* activity than cephalosporins, reduced susceptibility to these agents indicates that therapeutic options are becoming progressively restricted. Similar observations have been reported from neighboring countries, where amikacin frequently remains one of the more active aminoglycosides despite increasing resistance trends [40]. Likewise, carbapenems continue to demonstrate better activity than many β-lactams in several regional studies [35],[41]. Nevertheless, resistance patterns remain highly heterogeneous across geographical regions and healthcare settings, with some intensive-care cohorts reporting substantially higher carbapenem resistance rates [42]. Such variability most likely reflects differences in antimicrobial stewardship practices, local resistance ecology, infection-prevention measures, and patient case mix rather than intrinsic biological differences between circulating *K. pneumoniae* populations.

Resistance to fluoroquinolones and trimethoprim–sulfamethoxazole further illustrates the cumulative impact of prolonged antimicrobial selection pressure. Comparable resistance profiles have been documented throughout South Asia and the Middle East, where extensive use of fluoroquinolones has contributed to widespread dissemination of resistant Enterobacterales [43],[44]. In contrast, nitrofurantoin retained moderate activity among urinary isolates, supporting other observations that it remains an appropriate treatment option for selected uncomplicated urinary tract infections caused by susceptible isolates [35],[45]. The preserved activity of colistin and tigecycline against most isolates is encouraging; however, these agents represent last-line therapeutic options whose increasing use may accelerate the emergence of additional resistance mechanisms. Consequently, reliance on these drugs should be accompanied by robust antimicrobial stewardship and continuous surveillance to preserve their clinical utility [42],[46],[47].

The overall burden of multidrug resistance observed in this study is consistent with the growing regional and global expansion of MDR and XDR *K. pneumoniae* reported from Pakistan and neighboring countries [36],[48],[49]. Rather than representing isolated resistance events, these findings likely reflect the cumulative effects of sustained antimicrobial selective pressure, horizontal transfer of resistance determinants, and successful dissemination of internationally recognized high-risk clones. Collectively, these observations reinforce the need for integrated phenotypic and genomic surveillance to support evidence-based infection prevention and antimicrobial stewardship programs [50],[51].

An important finding of this study is the discordance between phenotypic hypermucoviscosity and genomic virulence profiles. Although a proportion of isolates exhibited a positive string test, whole-genome sequencing demonstrated a consistently low virulence background, with no detection of the canonical hypervirulence-associated loci, including *iuc*, *clb*, *rmpA*, or *rmpA2*. Instead, yersiniabactin represented the only virulence determinant detected and was identified across genetic backgrounds through different ICEKp lineages. These findings indicate that the circulating isolates are more appropriately classified as non-hypervirulent MDR *K*. *pneumoniae* clones. This distinction is clinically important because hypermucoviscosity alone is no longer considered a reliable surrogate marker of hypervirulence, and genuinely hypervirulent isolates may not necessarily exhibit a hypermucoviscous phenotype [37],[52]. Our findings therefore support the growing consensus that accurate characterization of virulence requires genomic confirmation rather than reliance on phenotypic assays alone [53],[54].

WGS further demonstrated substantial genomic diversity among the study isolates despite the relatively limited number of genomes analyzed. The predominance of ST147 together with the identification of other internationally recognized high-risk lineages, including ST11 and ST231, is consistent with global reports describing these clones as successful epidemic lineages capable of accumulating antimicrobial resistance determinants while maintaining efficient nosocomial transmission [55],[56]. The close association between ST147 and the KL64 capsular locus observed in this study is likewise consistent with previous genomic investigations [57]. However, unlike reports describing convergence of resistance and hypervirulence within certain ST147 populations, particularly those reported from Italy, the ST147 isolates characterized here lacked the principal hypervirulence-associated loci despite carrying extensive resistance determinants [58]. These observations suggest that the regional success of ST147 in our setting is primarily attributable to antimicrobial resistance and clonal dissemination rather than acquisition of the hypervirulence plasmid characteristic of convergent CR-hvKP strains.

The resistome identified by WGS closely mirrors the molecular epidemiology reported throughout South Asia. The predominance of *bla*CTX-M-15 together with the presence of *bla*CTX-M-14 supports the continued dominance of CTX-M-type ESBLs in *K. pneumoniae* circulating in Pakistan [59]–[62]. Likewise, the detection of *bla*NDM-5, *bla*OXA-48, and *bla*OXA-181 confirms the ongoing dissemination of clinically important carbapenemases within the region [12],[63]. Rather than occurring independently, these acquired resistance genes frequently coexisted with chromosomal mutations affecting fluoroquinolone targets, porin-associated permeability, and colistin susceptibility, illustrating the multifactorial nature of antimicrobial resistance evolution in *K. pneumoniae*. This combination of plasmid-mediated and chromosomal resistance mechanisms substantially limits therapeutic options and highlights the adaptive capacity of successful hospital-associated lineages [64]–[67].

Phylogenomic analysis demonstrated that the study isolates clustered within globally distributed clonal lineages rather than forming a distinct regional population. Their close relationship to other reported Pakistani genomes supports continued circulation of established lineages within the country, whereas their phylogenetic proximity to genomes originating from Asia, the Middle East, and Europe is consistent with the international dissemination of successful epidemic clones [55],[68]. However, because only a limited number of genomes from a single geographical region were analyzed and epidemiological linkage data were unavailable, these findings should be interpreted as evidence of shared evolutionary ancestry rather than direct transmission events or recent international dissemination. Higher-resolution approaches, including core-genome SNP analyses combined with epidemiological investigations, would provide greater resolution for reconstructing transmission dynamics in future studies.

This study possesses several strengths, including the integration of phenotypic antimicrobial susceptibility testing with WGS and comparative genomic analyses to characterize clinically important *K. pneumoniae* lineages from multiple tertiary-care hospitals. Nevertheless, several limitations should be acknowledged. First, we employed an observational cross-sectional design without formal statistical comparisons between genomic lineages; therefore, associations between sequence types, resistance determinants, and clinical characteristics should be interpreted descriptively. Second, although isolates were collected from three major referral hospitals, the sampling strategy reflected routine clinical submissions rather than systematic population-based surveillance, resulting in predominance of urine and pus specimens and comparatively fewer bloodstream isolates. Consequently, the observed lineage distribution may not fully represent the broader epidemiology of *K. pneumoniae* across all infection types or healthcare settings. Third, WGS was performed on a purposive subset of MDR/XDR isolates rather than the complete isolate collection, limiting the ability to estimate lineage prevalence at the population level. Furthermore, following quality-control procedures, nine sequencing datasets were excluded because of species misclassification or evidence of contamination, leaving 34 high-quality genomes for comparative analyses. Although these exclusions strengthened the reliability of downstream genomic analyses, they reduced the overall genomic sample size. Finally, phylogenetic inference was based on conserved core genes rather than high-resolution core-genome SNP analysis; therefore, this study was designed to evaluate population structure and evolutionary relationships rather than infer direct transmission events.

Despite these limitations, this study provides one of the most comprehensive genomic characterizations of clinical *K. pneumoniae* available from northwestern Pakistan. The findings demonstrate that MDR *K. pneumoniae* circulating in tertiary-care hospitals are dominated by internationally disseminated high-risk lineages possessing extensive antimicrobial resistance determinants but comparatively limited virulence gene repertoires. Continued implementation of integrated genomic surveillance, supported by expanded sequencing efforts and standardized epidemiological investigations, will be essential for monitoring the emergence of high-risk clones, guiding infection prevention strategies, and strengthening antimicrobial stewardship programs in Pakistan and comparable resource-limited settings.

## 5. Conclusion

This study provides comprehensive phenotypic and whole-genome sequencing-based insights into the molecular epidemiology of clinical *K. pneumoniae* isolates circulating in tertiary-care hospitals in Peshawar, Pakistan. The findings demonstrate substantial genetic diversity among MDR and XDR isolates, with predominance of internationally recognized high-risk lineages, particularly ST147, harboring antimicrobial resistance determinants, including extended-spectrum β-lactamase and carbapenemase genes, but a comparatively limited repertoire of canonical hypervirulence-associated genes. These observations suggest that the regional population is characterized primarily by the dissemination of antimicrobial-resistant classical *K. pneumoniae* lineages rather than widespread convergence of resistance and hypervirulence. Although phylogenomic analyses revealed genetic relatedness between the study isolates and previously reported national and international genomes, the cross-sectional design and limited number of sequenced isolates do not permit definitive conclusions regarding transmission pathways or evolutionary dynamics. Instead, these findings establish an important genomic baseline for future longitudinal surveillance incorporating larger collections of clinical isolates, high-resolution phylogenomic analyses, and detailed epidemiological data to better understand the emergence, persistence, and dissemination of high-risk *K. pneumoniae* clones in Pakistan. Collectively, this study highlights the value of integrating whole-genome sequencing into routine antimicrobial resistance surveillance programs to support evidence-based infection prevention, antimicrobial stewardship, and public health interventions aimed at limiting the spread of clinically important MDR *K. pneumoniae*.

## Use of AI tools declaration

The authors state that they have not utilized Artificial Intelligence (AI) tools when creating this article.



## References

[1] Aldali JA (2025). *Klebsiella pneumoniae*: A multidrug-resistant pathogen has emerged in Saudi Arabia. Front Microbiol.

[2] Huang X, Yao X, Hou Y (2025). Global trends of antimicrobial resistance and virulence of *Klebsiella pneumoniae* from different host sources. Commun Med.

[3] García-Cobos S, Oteo-Iglesias J, Pérez-Vázquez M (2025). Hypervirulent *Klebsiella pneumoniae*: Epidemiology outside Asian countries, antibiotic resistance association, methods of detection and clinical management. Enferm Infecc Microbiol Clin.

[4] Choby JE, Howard-Anderson J, Weiss DS (2020). Hypervirulent *Klebsiella pneumoniae*—clinical and molecular perspectives. J Intern Med.

[5] Wang Q, Ye MY, Hong C (2025). The mechanisms of resistance, epidemiological characteristics, and molecular evolution of carbapenem-resistant hypervirulent *Klebsiella pneumoniae*. Lab Med.

[6] Chen TA, Chuang YT, Lin CH (2024). A decade-long review of the virulence, resistance, and epidemiological risks of *Klebsiella pneumoniae* in ICUs. Microorganisms.

[7] Le Bris J, Chen N, Supandy A (2025). Phage therapy for *Klebsiella pneumoniae*: Understanding bacteria–phage interactions for therapeutic innovations. PLoS Pathog.

[8] Stanton TD, Keegan SP, Abdulahi JA (2026). Distribution of capsule and O types in *Klebsiella pneumoniae* causing neonatal sepsis in Africa and South Asia: A meta-analysis of genome-predicted serotype prevalence to inform potential vaccine coverage. PLoS Med.

[9] Karampatakis T, Tsergouli K, Behzadi P (2023). Carbapenem-resistant *Klebsiella pneumoniae*: Virulence factors, molecular epidemiology and latest updates in treatment options. Antibiotics.

[10] Mishra P, Sahoo D, Sahu MC (2025). Genetic diversity and antimicrobial resistance of clinical *Klebsiella pneumoniae* isolates: An ISSR-PCR analysis. J Infect Public Health.

[11] Spadar A, Perdigão J, Campino S (2023). Large-scale genomic analysis of global *Klebsiella pneumoniae* plasmids reveals multiple simultaneous clusters of carbapenem-resistant hypervirulent strains. Genome Med.

[12] Maguire M (2025). Carbapenem resistance and hypervirulence in Enterobacterales: A genomic and phylogenetic analysis of clinical and environmental isolates in Ireland.

[13] El Sharief H, Ali SM, Mohamed S (2025). Prevalence of carbapenemase genes *blaOXA*-48, *bla*NDM, and *bla*IMP in multidrug-resistant *Pseudomonas aeruginosa* and *Klebsiella pneumoniae* clinical isolates from University Hospital Sharjah, UAE. Int J Biomedicine.

[14] Zhang Z, Wang D, Li Y (2022). Comparison of the performance of phenotypic methods for the detection of carbapenem-resistant Enterobacteriaceae (CRE) in clinical practice. Front Cell Infect Microbiol.

[15] Maryam S, Saleem Z, Haseeb A (2025). Progress on the global research agenda for antimicrobial resistance in human health in Pakistan: Findings and implications. Infect Drug Resist.

[16] Ashraf Z, Hidayat Rasool M, Khurshid M (2025). Antimicrobial resistance and virulence profiling of *Klebsiella pneumoniae* isolated from urinary tract infections in community and hospital settings in Pakistan. Pak J Pharm Sci.

[17] Rizvi M, Khan N, Fatima A (2025). The microbiological characteristics and genomic surveillance of carbapenem-resistant *Klebsiella pneumoniae* isolated from clinical samples. Microorganisms.

[18] Somda NS, Nyarkoh R, Kotey FCN (2024). A systematic review and meta-analysis of carbapenem-resistant Enterobacteriaceae in West Africa. BMC Med Genomics.

[19] Haley E, Cockerill FR, Pesano RL (2024). Pooled antibiotic susceptibility testing performs within CLSI standards for validation when measured against broth microdilution and disk diffusion antibiotic susceptibility testing of cultured isolates. Antibiotics.

[20] Smriti S, Panda SS, Dash RK (2025). Antibiogram of *Escherichia coli* and *Klebsiella pneumoniae* urinary isolates and susceptibility of amikacin in extended-spectrum beta-lactamase producers. J Pure Appl Microbiol.

[21] Narayan M, Mohapatra S, Das B (2025). Rapid identification and susceptibility testing directly from urine specimens, reducing turnaround time, a step towards advancing diagnostic stewardship. Cureus.

[22] Magiorakos AP, Srinivasan A, Carey RB (2012). Multidrug-resistant, extensively drug-resistant and pandrug-resistant bacteria: An international expert proposal for interim standard definitions for acquired resistance. Clin Microbiol Infect.

[23] Thapa TB, Maharjan S, Giri N (2025). Incidence of hypermucoviscous *Klebsiella pneumoniae* and phenotypic detection of their virulence factors along with classical strains among patients visiting tertiary care hospital. J Infect Dev Countr.

[24] Lad V, Panchal D, Pithawala M (2022). Determination of hemolytic activity. Biosafety Assessment of Probiotic Potential.

[25] Quainoo S, Coolen JPM, van Hijum SAFT (2017). Whole-genome sequencing of bacterial pathogens: The future of nosocomial outbreak analysis. Clin Microbiol Rev.

[26] Sewe SO, Silva G, Sicat P (2022). Trimming and validation of Illumina short reads using trimmomatic, trinity assembly, and assessment of RNA-Seq data. Plant Bioinformatics. Methods in Molecular Biology.

[27] Wood DE, Lu J, Langmead B (2019). Improved metagenomic analysis with Kraken 2. Genome Biol.

[28] Liang H, Zou Y, Wang M (2025). Efficiently constructing complete genomes with CycloneSEQ to fill gaps in bacterial draft assemblies. GigaByte.

[29] Faruk MO, Sarkar MMH, Hossain MDI (2024). Genomic characterization and functional insights of multidrug-resistant *Klebsiella pneumoniae* strain BCSIR-JUMIID. bioRxiv.

[30] Sayem M, Rafi MA, Mishu ID (2025). Comprehensive genomic analysis reveals virulence and antibiotic resistance genes in a multidrug-resistant *Bacillus cereus* isolated from hospital wastewater in Bangladesh. Sci Rep.

[31] Azra, Khan TA, Ul Haq I (2025). Antibiotic susceptibility patterns and virulence profiles of classical and hypervirulent *Klebsiella pneumoniae* strains isolated from clinical samples in Khyber Pakhtunkhwa, Pakistan. Pathogens.

[32] Sah RSP, Dhungel B, Yadav BK (2021). Detection of *TEM* and *CTX-M* genes in *Escherichia coli* isolated from clinical specimens at tertiary care heart hospital, Kathmandu, Nepal. Diseases.

[33] Bina M, Pournajaf A, Mirkalantari S (2015). Detection of the *Klebsiella pneumoniae* carbapenemase (KPC) in *K. pneumoniae* isolated from clinical samples by phenotypic and genotypic methods. Iran J Pathol.

[34] Pérez-Vázquez M, Oteo-Iglesias J, Sola-Campoy PJ (2019). Characterization of carbapenemase-producing *Klebsiella oxytoca* in Spain, 2016–2017. Antimicrob Agents Chemother.

[35] Kuinkel S, Acharya J, Dhungel B (2021). Biofilm formation and phenotypic detection of ESBL, MBL, KPC and AmpC enzymes and their coexistence in *Klebsiella* spp. isolated at the National Reference Laboratory, Kathmandu, Nepal. Microbiol Res.

[36] Nirwati H, Sinanjung K, Fahrunissa F (2019). Biofilm formation and antibiotic resistance of *Klebsiella pneumoniae* isolated from clinical samples in a tertiary care hospital, Klaten, Indonesia. BMC Proc.

[37] Hassan NM, Dawood AB, Mohamed MM (2024). Distribution, characterization and antibiotic resistance of hypervirulent *Klebsiella pneumoniae* (hvKp) strains versus classical strains (CKp) causing healthcare-associated infections in Sohag University Hospitals. Microbes Infect Dis.

[38] Moustafa NM, Mahmoud FM, Khamsin NWB (2023). Antimicrobial susceptibility of *Klebsiella pneumoniae* isolated from intensive and non-intensive care units patients: A one-year retrospective study in a tertiary healthcare hospital, Saudi Arabia. J Pure Appl Microbiol.

[39] Wani FA, Bandy A, Alenzi MJS (2021). Resistance patterns of gram-negative bacteria recovered from clinical specimens of intensive care patients. Microorganisms.

[40] Ramirez MS, Tolmasky ME (2017). Amikacin: Uses, resistance, and prospects for inhibition. Molecules.

[41] Kazmierczak KM, Karlowsky JA, de Jonge BLM (2021). Epidemiology of carbapenem resistance determinants identified in meropenem-nonsusceptible Enterobacterales collected as part of a global surveillance program, 2012 to 2017. Antimicrob Agents Chemother.

[42] Sader HS, Mendes RE, Streit JM (2022). Antimicrobial susceptibility of gram-negative bacteria from intensive care unit and non-intensive care unit patients from United States hospitals (2018–2020). Diagn Microbiol Infect Dis.

[43] Silvester R, Madhavan A, Kokkat A (2022). Global surveillance of antimicrobial resistance and hypervirulence in *Klebsiella pneumoniae* from LMICs: An in-silico approach. Sci Total Environ.

[44] Dalhoff A (2012). Global fluoroquinolone resistance epidemiology and implications for clinical use. Interdiscip Perspect Infect Dis.

[45] Munoz-Davila MJ (2014). Role of old antibiotics in the era of antibiotic resistance: Highlighting nitrofurantoin for the treatment of lower urinary tract infections. Antibiotics.

[46] Xiao S, Chen T, Wang H (2021). Drug susceptibility and molecular epidemiology of *Klebsiella pneumoniae* bloodstream infection in ICU patients in Shanghai, China. Front Med.

[47] Bassetti M, Vena A, Sepulcri C (2020). Treatment of bloodstream infections due to gram-negative bacteria with difficult-to-treat resistance. Antibiotics.

[48] Mohd Asri NA, Ahmad S, Mohamud R (2021). Global prevalence of nosocomial multidrug-resistant *Klebsiella pneumoniae*: A systematic review and meta-analysis. Antibiotics.

[49] Nakamura-Silva R, Cerdeira L, Oliveira-Silva M (2022). Multidrug-resistant *Klebsiella pneumoniae*: A retrospective study in Manaus, Brazil. Arch Microbiol.

[50] Abbott IJ, Peel TN, Cairns KA (2023). Antibiotic management of urinary tract infections in the post-antibiotic era: A narrative review highlighting diagnostic and antimicrobial stewardship. Clin Microbiol Infect.

[51] Kot B, Piechota M, Szweda P (2023). Virulence analysis and antibiotic resistance of *Klebsiella pneumoniae* isolates from hospitalised patients in Poland. Sci Rep.

[52] Alharbi MT, Almuhayawi MS, Nagshabandi MK (2023). Antimicrobial resistance pattern, pathogenicity and molecular properties of hypervirulent *Klebsiella pneumoniae* (hvKp) among hospital-acquired infections in the intensive care unit (ICU). Microorganisms.

[53] Ali F (2024). Investigation of the resistance of *Klebsiella* bacteria to antibiotics and the synthesis of virulence factors isolated from various pathogenic infections. J La Lifesci.

[54] Ali H, Jabir DM, Falih Z (2025). Phenotypic and genotypic analysis of hypermucoviscous *Klebsiella pneumoniae* (hmKp) strain. Iran J Microbiol.

[55] Arcari G, Carattoli A (2023). Global spread and evolutionary convergence of multidrug-resistant and hypervirulent *Klebsiella pneumoniae* high-risk clones. Pathog Glob Health.

[56] Elias R, Phelan JE, Lito L (2025). Genome-wide analysis and longitudinal study of *Klebsiella pneumoniae* in Portugal: Tracing the evolution and spread of carbapenem resistance. Int J Antimicrob Agents.

[57] Al Fadhli AH, Mouftah SF, Jamal WY (2023). Cracking the code: Unveiling the diversity of carbapenem-resistant *Klebsiella pneumoniae* clones in the Arabian Peninsula through genomic surveillance. Antibiotics.

[58] Di Pilato V, De Angelis LH, Aiezza N (2022). Resistome and virulome accretion in an NDM-1-producing ST147 sublineage of *Klebsiella pneumoniae* associated with an outbreak in Tuscany, Italy: A genotypic and phenotypic characterisation. Lancet Microbe.

[59] Alam M, Saleem Z, Haseeb A (2023). Tackling antimicrobial resistance in primary care facilities across Pakistan: Current challenges and implications for the future. J Infect Public Health.

[60] Maveke SM, Aboge GO, Kanja LW (2024). Phenotypic and genotypic characterization of extended spectrum beta-lactamase-producing clinical isolates of *Escherichia coli* and *Klebsiella pneumoniae* in two Kenyan facilities: A national referral and a level five hospital. Int J Microbiol.

[61] Nossair MA, Abd El Baqy FA, Rizk MSY (2022). Prevalence and molecular characterization of extended-spectrum β-lactamases and AmpC β-lactamase-producing Enterobacteriaceae among human, cattle, and poultry. Pathogens.

[62] Shawa M, Chambaro H, Kamboyi HK (2025). In silico characterization of chromosomally integrated *bla*CTX-M genes among clinical Enterobacteriaceae in Africa: Insights from whole-genome analysis. Front Microbiol.

[63] Mitra S, Naha S, Chakraborty J (2025). Diversity of mobile genetic elements in carbapenem-resistant Enterobacterales isolated from the intensive care units of a tertiary care hospital in Northeast India. Front Microbiol.

[64] Gomaa Elsayed A, M Fahmy E, Abdellatif Alsayed M (2024). Study of plasmid-mediated quinolone resistance genes among *Escherichia coli* and *Klebsiella pneumoniae* isolated from pediatric patients with sepsis. Sci Rep.

[65] Vieira DC, Lima WG, de Paiva MC (2020). Plasmid-mediated quinolone resistance (PMQR) among Enterobacteriales in Latin America: A systematic review. Mol Biol Rep.

[66] Ahmed OB, Asghar AH, Bamaga M (2023). Characterization of aminoglycoside resistance genes in multidrug-resistant *Klebsiella pneumoniae* collected from tertiary hospitals during the COVID-19 pandemic. PLoS One.

[67] Zhang X, Li Q, Lin H (2021). High-level aminoglycoside resistance in human clinical *Klebsiella pneumoniae* complex isolates and characteristics of *armA*-carrying IncHI5 plasmids. Front Microbiol.

[68] Wyres KL, Lam MMC, Holt KE (2020). Population genomics of *Klebsiella pneumoniae*. Nat Rev Microbiol.

