# Supplementary material for: Genomic epidemiology of multidrug-resistant Klebsiella pneumoniae reveals lineage-specific resistance patterns with limited virulence determinants
Source: AIMS Microbiol. 2026 Sep 4;12(3):610–31. doi: 10.3934/microbiol.2026027 (PMC13652745; doi:10.3934/microbiol.2026027)
Supplement: Supplementary file 1 [file microbiol-12-03-027-s001.pdf]

AIMS Microbiology, 12(3): 610–631.
DOI: 10.3934/microbiol.2026027
Received: 27 February 2026
Revised: 28 July 2026
Accepted: 01 September 2026
Published: 04 September 2026

https://www.aimspress.com/journal/microbiology

Research article

Genomic epidemiology of multidrug-resistant *Klebsiella pneumoniae* reveals lineage-specific resistance patterns with limited virulence determinants

Aiman Waheed[1], Sumera Afzal Khan[1,*], Taj Ali Khan[2], Sajjad Ahmad[2], Aakash Ahmad Khattak[1], Taane G. Clark[3,4], Nigel G. Ternan[5], Fahad Ahmed[5], Ibrahim M. Banat[6,*] and Zahoor Khan[5]

[1] Center of Biotechnology and Microbiology, University of Peshawar, Peshawar, Pakistan
[2] Institute of Pathology and Diagnostic Medicine, IPDM, Khyber Medical University, Peshawar, Pakistan
[3] Department of Infection Biology, Faculty of Infectious and Tropical Diseases, London School of Hygiene and Tropical Medicine, London, UK
[4] Faculty of Epidemiology and Population Health, London School of Hygiene and Tropical Medicine, London, UK
[5] Nutrition Innovation Centre for Food and Health (NICHE), School of Biomedical Sciences, Ulster University, Coleraine, N. Ireland, UK
[6] Pharmaceutical Sciences Research Group, School of Biomedical Sciences, Ulster University, Coleraine, N. Ireland, UK

* **Correspondence:** Email: im.banat@ulster.ac.uk; drsumera@uok.edu.pk.

Supplementary

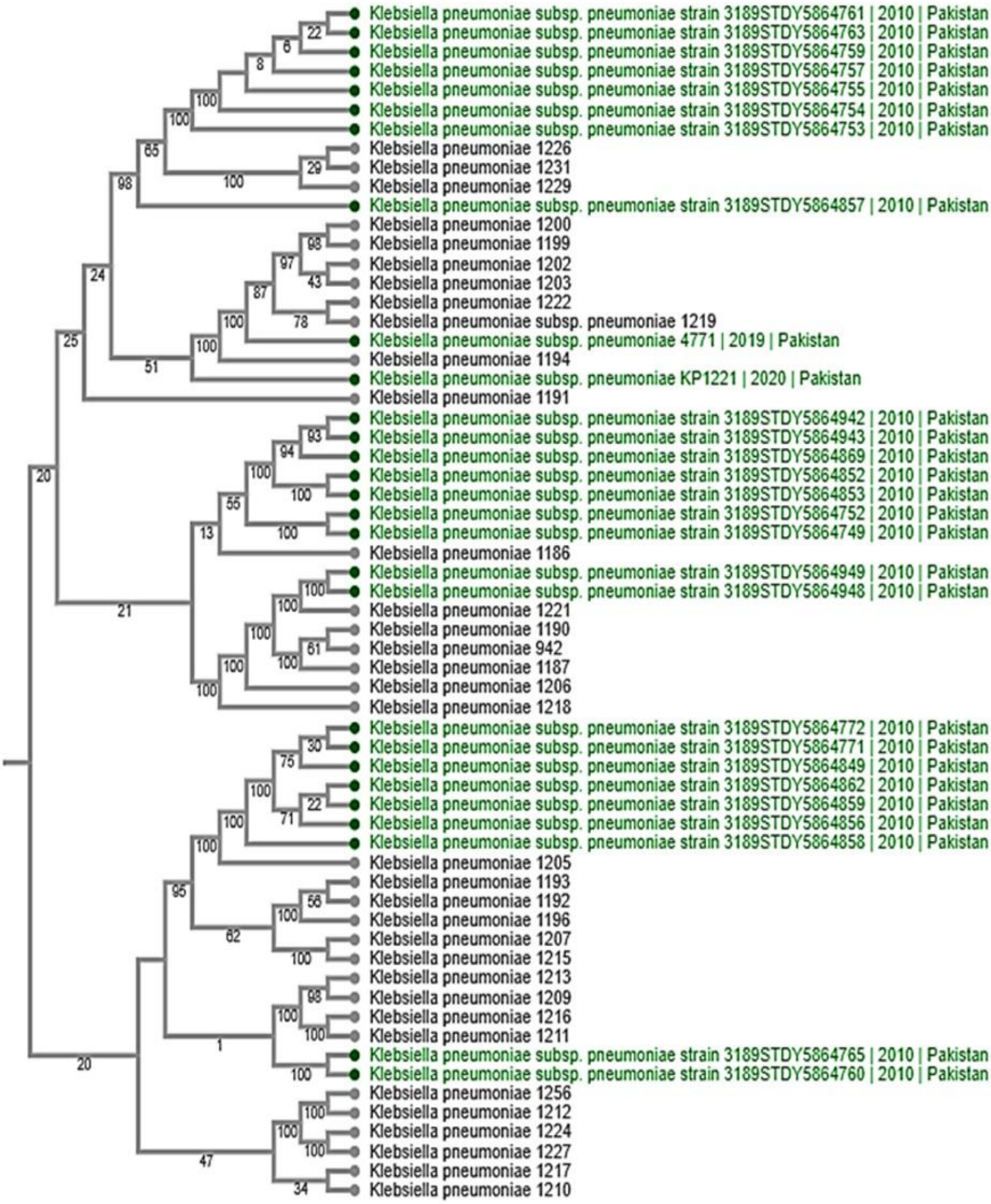

Figure S1. Maximum likelihood phylogenetic tree of *K. pneumoniae* isolates. Our study genomes are labeled in black, while publicly available Pakistan genomes retrieved from BV-BRC collected between 2010 and 2020 are labeled in green.

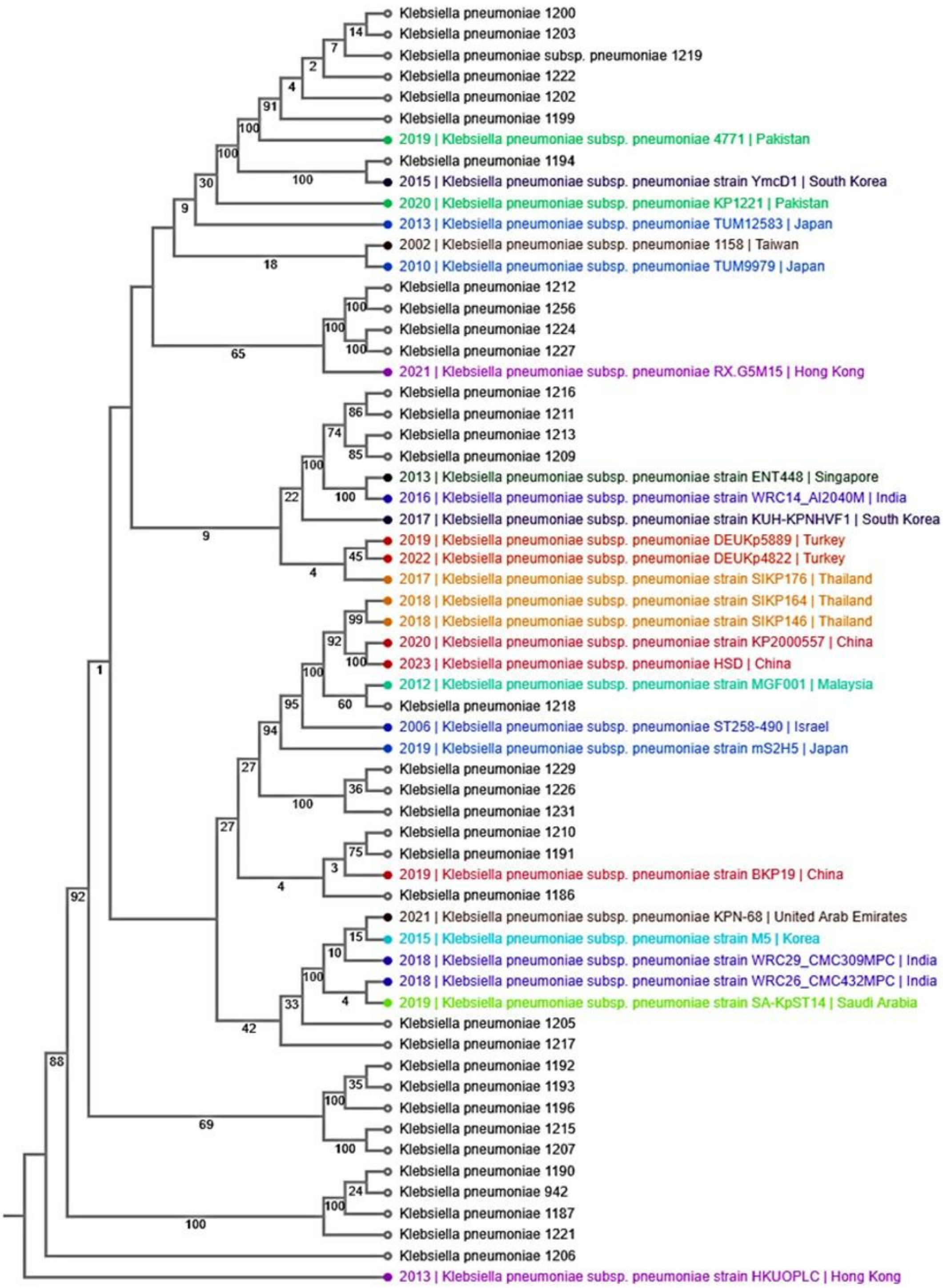

Figure S2. The phylogenomics tree shows the evolutionary relationship of our study *K. pneumoniae* genomes with global isolates. Our study genomes are shown in black, and publicly available global genomes retrieved from the BV-BRC are color-coded according to geographic origin.

AIMS Press

© 2026 the Author(s), licensee AIMS Press. This is an open access article distributed under the terms of the Creative Commons Attribution License (https://creativecommons.org/licenses/by/4.0)
